# General Principles of Treatment of Wounds of the Knee-joint

**Published:** 1918-12

**Authors:** 


***The General Principles of Treatment of Wounds of the Knee-Joint from Projectiles in Warfare.*** By CHARLES GREENE CUMSTON, M. D. *Annals of Surgery,* February, 1918.

Wounds of the knee-joint represent from 3 to 4 o/o of major injuries inflicted in battle, and the resulting mortality ranges around 18 per cent.

The knee-joint is particularly exposed to severe complications such as : secondary hemorrhage thrombophlebitis, gas gangrene, and sepsis.

The author considers that amputation is resorted to much too often. Amputation must be practised unquestionably in the following cases :

When there is severe crushing of the joint.

When the large vessels in the popliteal space are injured ; — attempts to save the limb in that case generally prove unsuccessful.

When there is gangrene of the leg, or when a purulent arthritis is complicated by secondary hemorrhage from the popliteal artery.

The most important thing to do is to disinfect the wound and then obtain thorough drainage and immobilization of the joint. The disinfection and drainage are best secured by arthrotomy. The author recommends either the U-shaped incision or a long external incision.

If arthrotomy does not seem sufficient, typical subperiosteal resection according to Ollier's technique, or a typical resection if an important portion of the articular surface can be preserved, should be practised within 12 or 24 hours of injury. Never suture the wound after resection, advises the author; leave it wide open without a single stitch being taken.

The different injuries of the knee that are most frequently dealt with may be classified as follows :

Tunnel wounds without fracture.

Wounds with fracture of one of the bones composing the joint.

Wounds with fracture of both tibia and femur.

Wounds with crushing of the articular surfaces, often extending to the surrounding soft parts.

1) *Penetrating Wounds without Lesions of the Bones.*

*a*) The projectile passing through the joint.

Simple capsular damages are not infrequent. When seen within the first 24 hours after receipt of the injury, the knee is already swollen and an entrance and exit wound will be found diametrically opposite each other. The patient may be either apyretic or feverish.

When there is apyrexia, rest in bed, compression and immobilization will generally prove efficient. If after three or four days the intra-articular fluid collection has not become absorbed, it is advisable to empty the joint by means of a small incision, followed by irrigation with salt solution. The incision is sutured with catgut, and a compressive dressing is applied which should not be removed for 5 or 6 days.

When there is pyrexia, expectant treatment would be a mistake. Arthrotomy should be practised without delay. An incision should be made on each side of the patella and a drain inserted into each. The limb should be immobilized in a posterior plaster case and elevated. The joint is washed once or twice daily with sterile salt solution, or some *very mild* antiseptic, after which a good practice is to spray the joint cavity with ether. As soon as the fever has disappeared, irrigations should be stopped, the incisions sutured, and aseptic dry dressings applied every 2 or 3 days.

*b*) The projectile remained in the wound.

A radiographic examination is necessary before undertaking any operation. It will show the exact position of the projectile, and also the bone lesions, if any exist.

The removal of the projectile must be undertaken as soon as possible; the joint is completely opened by suppressing the upper and lower capsular culs-de-sac. An inward dislocation of the patella is often advisable to render more easy the exploration of all the bones of the joint.

If the joint is infected the U-shaped incision proves most convenient to freely expose and drain the wound.

2) *Penetrating Wounds of the Knee with Bone Lesions.*

The extent of the damages done to the bones may be very variable. They may involve the tibia, or patella, or the lower end of the femur. Besides the principal lesions of the epiphyses, fissures may exist in the corresponding diaphysis; these fissures are most important factors in the development of osteomyelitis. Surrounding soft parts may also be damaged, and bring forward serious complications.

Arthrotomy is insufficient in all the severe cases; the author considers that if arthrotomy only be practised, osteomyelitis will nearly always follow necessarily. In cases in which arthrotomy has given good results, he ascertains that the lesions were confined to the synovia, and no damage done to the articular surfaces.

The proper treatment in these cases, when seen within the first 36 hours, is typical subperiosteal resection if the amount of damage done does not require the excision of more than 10 cm., as otherwise the resulting shortening would leave a useless limb.

If amputation must be resorted to, the author advises performing circular amputation as low down on the thigh as possible, and never suturing the stump. The further development of infection will thus be avoided.

